# Concepts of Regeneration for Spinal Diseases in 2023

**DOI:** 10.3390/ijms242216335

**Published:** 2023-11-15

**Authors:** Takashi Yurube, Inbo Han, Daisuke Sakai

**Affiliations:** 1 Department of Orthopaedic Surgery, Graduate School of Medicine, Kobe University, 7-5-1 Kusunoki-cho, Chuo-ku, Kobe 650-0017, Japan; 2 Department of Neurosurgery, CHA University School of Medicine, CHA Bundang Medical Center, Seongnam-si 13496, Republic of Korea; hanib@cha.ac.kr; 3 Department of Orthopedic Surgery, School of Medicine, Tokai University, 143 Shimokasuya, Isehara 259-1193, Japan; daisakai@is.icc.u-tokai.ac.jp

## 1. Introduction

It is our pleasure to announce the publication of the Special Issue “Regeneration for Spinal Diseases 3.0” in the *International Journal of Molecular Sciences* (ISSN 1422-0067). Spinal diseases place a significant physical, mental, and social burden on the general population [1]. As the morbidity of degenerative spinal pathologies, including intervertebral disc disorder and osteoporosis, is quite high and increases with age, the affected population may suffer from a long-term disability [2]. Furthermore, traumatically or non-traumatically, the most serious spine-related conditions can ultimately result in spinal cord injury [3]. Despite recent advances in the management of these prevalent but problematic spinal diseases, there is a growing research interest in discovering novel therapeutic strategies.

Our previous *International Journal of Molecular Sciences* Special Issues, “Regeneration for Spinal Diseases” indexing 15 articles in 2021 [4] and “Regeneration for Spinal Diseases 2.0” indexing 14 articles in 2022 [5] were both successful because of the publication of many high-quality papers with rigorous scientific backgrounds. Now, in 2023, we would like to add further results and new perspectives from more recent research projects to the 1st and 2nd editions. This 3rd *International Journal of Molecular Sciences* Special Issue, “Regeneration for Spinal Diseases 3.0”, includes nine cutting-edge original research articles, consisting of six papers for degenerative disc disease, one for spinal cord injury, one for spinal muscular atrophy (SMA), and one for spinal fusion surgery. Moreover, one expertized review articles summarizing the treatment application of platelet-rich plasma (PRP) for spinal problems. All 10 articles should provide helpful insights for the current clinical management and future basic scientific development of regenerative treatment strategies for intractable spinal diseases.

These articles were published in the *International Journal of Molecular Sciences* (https://www.mdpi.com/journal/ijms, accessed on 1 November 2023), Section: Molecular Pathology, Diagnostics, and Therapeutics (https://www.mdpi.com/journal/ijms/sections/Pathology_Diagnostics_Therapeutics, accessed on 1 November 2023), Topic: Regeneration for Spinal Diseases 3.0 (https://www.mdpi.com/journal/ijms/special_issues/MZN0W0CUIU, accessed on 1 November 2023). Articles in the previous edition of this Special Issue were also published in the *International Journal of Molecular Sciences*, Section: Molecular Pathology, Diagnostics, and Therapeutics, Topic: Regeneration for Spinal Diseases (https://www.mdpi.com/journal/ijms/special_issues/Spinal_Diseases, accessed on 1 November 2023) and Regeneration for Spinal Diseases 2.0 (https://www.mdpi.com/journal/ijms/special_issues/Spinal_Diseases_2, accessed on 1 November 2023). The *International Journal of Molecular Sciences* is an international, peer-reviewed, open access journal published by the Multidisciplinary Digital Publishing Institute, universally and freely accessible online.

## 2. Degenerative Disc Disease

With the global trend of aging, back pain is a worldwide health problem because of the enormous morbidity and socioeconomic strain [1,6,7]. The cause of back pain is largely non-specific [8]; however, intervertebral disc degeneration is an independent risk factor [9]. Degenerative disc disease increases with age [10], which can develop not only severe nociceptive back and neuropathic arm and leg pain but also neurological radiculopathy and myelopathy, including paralysis, intermittent claudication, and even bladder and bowel dysfunction [11]. The current primary treatment for degenerative disc disease is surgical resection and/or spinal fusion, resulting in the loss of load, shock absorption, and movement function [12]. Therefore, the development of regenerative therapies for intervertebral disc degeneration is highly demanded.

The intervertebral disc has a complex structure, consisting of the central nucleus pulposus (NP), surrounding annulus fibrosus (AF), and sandwiching cartilage endplates [11]. The disc is immune-privileged and also the largest avascular, hypoxic, low-nutrient organ in the body [13]. Particularly, in the NP compared to the AF, cells depend on the diffusion from blood vessels at the disc margins to obtain nutrients [13]. Thus, reduced blood supply, subchondral bone sclerosis, and endplate calcification, all occurring with mechanical stress, injury, smoking, radiation, and aging, can limit the transport of nutrients to the disc easily [13]. This additional nutrient deprivation is a suspected contributor to intervertebral disc degeneration, which subsequently facilitates the development of symptomatic degenerative disc disease.

Kawaguchi K et al. confirmed gene and protein expression of growth arrest and DNA damage 45 gamma (GADD45G) [14] and cytoplasmic activation/proliferation-associated protein-1 (CAPRIN1) [15] in human degenerative discs with magnetic resonance imaging (MRI) grades 2–4 [16]. These molecules were based on prior evidence [17]. The percentage of immunopositive cells for GADD45G and CAPRIN1 both increased with the severity of MRI-based degeneration in human disc NP tissues. Then, the immunopositive cell percentage for GADD45G but not for CAPRIN1 correlated with the severity of histological degeneration. According to these findings, the expression of these two cell-cycle-related molecules, GADD45G and CAPRIN1, might be regulated during the progression of degeneration to maintain the integrity of human disc NP tissues by controlling cell proliferation and death under epigenetic alteration. Further mechanistic investigation is required.

Yu Y et al. reported the degeneration severity-dependent involvement of pyroptosis, an inflammatory form of lytic programmed cell death [18], in human intervertebral disc NP tissues. Next, in vitro treatment using exosomes derived from human embryonic stem cells (hESCs-exo) [19] successfully mitigated hydrogen peroxide-induced disc NP cell pyroptosis. Then, bioinformatics predicted that microRNA (miR)-302c, an embryonic stem cell-specific RNA abundant in hESCs-exo, would alleviate disc NP cell pyroptosis, which was further confirmed by the gain-of-function experiment. In vivo experiments also displayed that hESCs-exo and miR-302c could slow both histomorphological and biochemical annular puncture-induced degeneration through pyroptosis inhibition in rat tail discs. In summary, hESCs-exo-derived miR-302c might be useful to treat intervertebral disc degeneration and also symptomatic degenerative disc disease by regulating pyroptosis associated with the inflammatory response.

Kakutani K et al. studied the effects of cytokine inhibitors, interleukin-1 receptor antagonist (IL-1Ra), and soluble tumor necrosis factor receptor-1 (sTNFR1) [20], on human disc extracellular matrix metabolism. The simultaneous administration of IL-1Ra and sTNFR1 stimulated proteoglycan and collagen synthesis in human disc NP and AF cells in the alginate beads and tissue explants. The relative increase in proteoglycan synthesis correlated with the concentration of IL-1beta (IL-1β) in human disc AF explants after the co-administration of cytokine inhibitors. The relative increase in proteoglycan synthesis further correlated with the severity of degeneration assessed via MRI in human disc NP and AF explants after the simultaneous cytokine inhibitor treatment. Moreover, secretion levels of IL-1β and TNF-alpha (TNF-α) increased through the single administration of IL-1Ra and sTNFR1 but markedly decreased through the simultaneous administration of these cytokine inhibitors. Hence, IL-1Ra and sTNFR1 have the potential to enhance proteoglycan and collagen synthesis in human discs. IL-1β and TNF-α both have the feedback pathway to maintain the optimal secretion, resulting in the control of homeostasis in intervertebral disc explants.

Ohnishi H et al. aimed to elucidate the effects of AdipoRon, an agonist of adiponectin receptors [21], on the disc NP using an in vitro three-dimensional human cell culture system and an in vivo rat tail annular puncture-induced degeneration model. Adipose tissue is a large endocrine organ, which secretes metabolic factors known as adipokines [22]. Adiponectin, an adipokine hormone secreted by adipocytes, has anti-diabetic, anti-atherogenic, and anti-inflammatory effects [22]. In vitro, IL-1β-induced upregulation of pro-inflammatory and catabolic gene expression was downregulated by AdipoRon in human disc NP cells. Then, AdipoRon enhanced adenosine monophosphate-activated protein kinase phosphorylation as well as suppressed NF-κB p65 phosphorylation under pro-inflammatory IL-1β stimulation. In vivo, the intradiscal administration of AdipoRon was effective in alleviating radiologic height loss, histomorphological degeneration, and elevated expression of extracellular matrix catabolic enzymes and pro-inflammatory cytokines in rat tail annular puncture-induced disc degeneration. AdipoRon is, thus, a promising anti-inflammatory agent that has the potential to mitigate the progression of intervertebral disc degeneration.

Munesada D et al. examined the potential of hyaluronic acid (HA) in mitigating dimethyl sulfoxide (DMSO)-induced cytotoxicity, caused by the generation of reactive oxygen species (ROS) [23], to develop off-the-shelf therapeutic products for intervertebral disc repair using NP cells. Based on the protection of chondrocytes against ROS [24], the addition of HA to a thawed cryopreservation medium demonstrated a higher rate of human disc NP cell proliferation and positivity for Tie2, a disc NP progenitor cell marker [25], and larger number of Tie2-positive cells, with a trend toward the suppression of oxidative stress. Hence, HA treatment appears to alleviate the cytotoxicity of DMSO by suppressing ROS generation. These findings highlight the ability of HA to maintain disc NP cell functionalities, suggesting the mixing of HA at the time of cell transplantation as a useful enhancement for the treatment of degenerative disc disease.

Bhujel B et al. illustrated the effectiveness of an injectable engineered cartilage gel utilizing human fetal cartilage-derived progenitor cells (hFCPCs) [26] on intervertebral disc repair in a rat tail nucleotomy model. Intradiscal implantation of the cartilage gel promoted the repair of degeneration, compared to hFCPCs or hFCPC-derived cartilage extracellular matrix alone, by alleviating radiological, histological, and biochemical damage with maintained structure, hydration, cellularity, phenotype (Tie2 and brachyury), and matrix integrity (aggrecan and collagen type II), suppressing matrix degradation (matrix metalloproteinase-13), inflammation (TNF-α, IL-1β, and inducible nitric oxide synthase), and pain (calcitonin gene receptor protein and von Frey test). Consequently, cartilage gel incorporating hFCPCs has far superior therapeutic potential than its individual components, suggesting further translation of the cartilage gel with hFCPCs to large animal models and human subjects.

## 3. Spinal Cord Injury

Spinal cord injury is a devastating condition resulting from damage to the spinal cord, primarily caused by trauma but also by vascular disease, infection, and tumors, which can induce temporary or permanent motor, sensory, and autonomic dysfunction and associated complications [3]. Currently, effective treatments are very limited, which are largely symptom relieving and deterioration preventing, e.g., corticosteroid administration (controversial), decompression, and/or stabilization, and rehabilitation [27]. Therefore, there is a great need to develop new therapeutic strategies for neurological recovery and neural tissue repair.

Jeong SY et al. clarified the effects of a combination therapy of resolvin D1 (RvD1), a specialized pro-resolving lipid mediator with anti-inflammatory and neuroprotective properties [28], with peripheral nerve-derived stem cell (PNSC) spheroids [29]. In a previous study of a spinal cord injury model [29], neurological recovery and neuronal regeneration but the limited anti-inflammatory capabilities of PNSC spheroids necessitated a combined therapeutic approach. In vitro analysis confirmed the anti-inflammatory activity and inhibitory effects of RvD1 on pro-inflammatory cytokine expression from rat PNSC spheroids. In vivo rat spinal cord injury analysis presented that the combination therapy outperformed monotherapies, exhibiting enhanced neuronal regeneration, macrophage recruitment reduction, and anti-inflammation. In conclusion, the combined application of RvD1 and PNSC spheroids may represent a novel therapeutic approach for spinal cord injury.

## 4. Spinal Muscular Atrophy

Spinal muscular atrophy is an autosomal-recessive inherited neurodegenerative disease, which is rare but the most common genetic disorder resulting in infant mortality, characterized by a loss of motor neurons and progressive muscle weakness and atrophy [30,31]. This disease is caused by biallelic mutations in the survival of motor neuron 1 (SMN1) gene encoding the SMN protein, required for the survival of motor neurons, whereas all patients retain SMN2, an almost identical copy gene. Therefore, understanding the molecular functions of SMN can provide insights into the pathogenic mechanisms and treatment of SMA [32,33].

Muinos-Buhl A et al. investigated the longer-term therapeutic potential of neurocalcin delta (Ncald)-antisense oligonucleotides (ASOs) via an additional intracerebroventricular bolus injection at postnatal day 28 in a low-dose SMN-ASO-treated severe SMA mouse model [34,35]. In a previous study [34], a presymptomatic intracerebroventricular injection of Ncald-ASOs at postnatal day 2 increased histological and electrophysiological SMA hallmarks at postnatal day 21, although Ncald-ASOs showed a shorter duration of action limiting a long-term benefit, contrary to SMN-ASOs. Two weeks after the injection of Ncald-ASOs, wild-type mice presented reduced NCALD in the brain and spinal cord, which were well tolerated. In a double-blinded preclinical study combining low-dose SMN-ASOs at postnatal day 1 with Ncald-ASOs at postnatal days 2 and 28, Ncald-ASO reinjection ameliorated the electrophysiological defects and denervation of neuromuscular junctions at 2 months. In addition, among a full battery of newly developed human NCALD-ASOs, an identified non-toxic and highly efficient NCALD-ASO69 downregulated NCALD in human inducible pluripotent stem cell-derived motor neurons, restoring cytoskeletal dynamics and neuronal activity. This additional protective effect opens the avenue for combination therapies with SMN-ASOs and NCALD-ASOs in patients with SMA.

## 5. Spinal Fusion Surgery

As a result of developmental, degenerative, and traumatic spinal disorders, severe spondylosis, spondylolisthesis, spondylolysis, spinal deformity, and spinal fracture can cause disabling back, arm, and leg symptoms [36]. Spinal fusion surgery is an intervention to stabilize segments, including discs and facet joints, as well as to decompress neural tissues, including the dural tube and nerve roots, ultimately providing acceptable clinical and radiological outcomes [37]. To achieve successful spinal fusion, the use of autologous bone graft is the gold standard [38]. However, due to the limitation of the supply and morbidity associated with autograft harvest [39], various bone graft materials, including allografts, demineralized bone matrix, synthetic bone substitutes consisting of ceramics and cements, and biological scaffolds, combined with growth factors, e.g., bone morphogenetic proteins (BMPs), have been developed [40].

Lee HY et al. tested the in vitro release kinetics from scaffold materials of recombinant human BMP-2 (rhBMP-2) [41], showing the hydroxyapatite (HA)/beta-tricalcium phosphate (β-TCP) hydrogel polymer mixture [42] as the optimal carrier for sustained release. Then, the in vivo analysis using a mini-pig spinal fusion model presented the substantial improvement by rhBMP-2 in the fusion rate and speed, with a trend toward a dose-dependent increase; however, the lower-dosage group demonstrated higher values, a reversed pattern over time. In addition, rhBMP-2 induced the formation of osteophytes, although it was not dose-dependent. In this study, the sustained release of rhBMP-2 by using the HA/β-TCP hydrogel carrier was successful, overcoming the drawback of potentially causing side effects when used at high concentrations. This system is efficient in terms of mineral matter to improve the spinal fusion rate and also bone quality.

## 6. Platelet-Rich Plasma

Platelet-rich plasma is a concentrate of PRP protein derived from whole blood, including blood plasma and autologous conditioned plasma, but centrifuged to remove red blood cells [43]. Consequently, PRP contains multiple types of growth factors, cytokines, and chemokines, which can exert regenerative potential for damaged tissues, e.g., tendon, ligament, and cartilage [44]. There are possible indications for the application of PRP to treat acute injuries and chronic diseases, even in spine surgery [45]. To understand the current and future benefits of PRP for the clinical treatment of spinal diseases, high-quality review articles are necessary.

Kawabata S et al. provided a comprehensive literature review article on the basic research progress and clinical application of PRP therapy for spinal disorders. In vitro and in vivo studies clarified the potential of PRP in repairing intervertebral disc degeneration, promoting spinal bone fusion, and facilitating neurological recovery from spinal cord injury. Clinical studies presented PRP’s analgesic effects on lower back and radicular pain and promoting effects on spinal bone fusion during surgery. Based on accumulating evidence in aspects of basic science and clinical practice, the efficacy and safety of PRP would be promising for spinal disease treatment with regenerative tissue repair [46]. Nevertheless, further randomized controlled trials, systematic reviews, and meta-analysis studies are warranted to develop safe, effective PRP therapy.

## 7. Conclusions

We would like to announce further regathering and recomposing for further successful, updated Special Issues, e.g., “Regeneration for Spinal Diseases 4.0”.
